# Use of Automated Insulin Delivery in Pregnancies Complicated by Type 1 Diabetes

**DOI:** 10.17925/EE.2024.20.2.14

**Published:** 2024-10-09

**Authors:** Mahima Chillakanti, Elaine Young, April Hopcroft, Natalie Bellini, Jennifer Smith, Diana Isaacs

**Affiliations:** 1. Close Concerns, San Francisco, CA, USA; 2. David Geffen School of Medicine at UCLA, Los Angeles, CA, USA; 3. The diaTribe Foundation, San Francisco, CA, USA; 4. University Hospitals, Cleveland, OH, USA; 5. Integrated Diabetes Services, Wynnewood, PA, USA; 6. Cleveland Clinic, Cleveland, OH, USA

**Keywords:** Automated insulin delivery, continuous glucose monitoring, glycaemic outcomes, insulin, pregnancy, time in range, type 1 diabetes

## Abstract

**Background:** Diabetes during pregnancy is associated with significant maternal and foetal health risks. Insulin requirements also change during pregnancy. This necessitates careful and effective management of diabetes. Although commonly used in clinical practice, the US Food and Drug Administration (FDA)-approved algorithms for automated insulin delivery (AID) systems do not have pregnancy-specific glycaemic targets. This review aims to evaluate the safety and efficacy of AID systems in reaching glycaemic targets in pregnant women with type 1 diabetes (T1D). **Methods:** In this retrospective case review, six pregnant women with T1D used three types of AID systems. Two patients used Omnipod 5, two patients used Control-I Q and two patients used Do-I t-Yourself (DIY) Loop. **Results:** Across trimesters, the two patients using Omnipod 5 had an average time in range (TIR) of 68 and 82%. Patients using Control-I Q had an average TIR of 77 and 69%. Both the patients using DIY Loop had an average TIR of 85%. Hypoglycaemia occurrence was minimal. Additionally, four of the six patients had uncomplicated vaginal deliveries in their third trimester, and four of the six patients achieved guideline-r ecommended TIR targets. Birth complications for the other two patients were resolved shortly after birth. Throughout the pregnancies, insulin needs approximately doubled. **Conclusions:** AID systems can achieve near-desired glycaemic targets with minimal hypoglycaemia in pregnant women with T1D. Randomized controlled trials are needed to confirm these findings and to win FDA indications in pregnancy.

The prevalence of diabetes during pregnancy is rapidly increasing. In the USA alone, an estimated 1–2% of pregnant women have type 1 diabetes (T1D) or type 2 diabetes (T2D), and an additional 6–9% develop gestational diabetes.^1^ From 2000 to 2010, the prevalence of gestational diabetes increased by 56%, and the prevalence of existing diabetes among pregnant women increased by 37%.^2^

Diabetes during pregnancy is associated with significant maternal and foetal health risks, typically relating to the degree of hyperglycaemia and chronic diabetes comorbidities.^3^ Adverse foetal outcomes include foetal and neonatal loss, congenital abnormalities, premature deliveries, macrosomia, neonatal hypoglycaemia and neonatal respiratory distress syndrome. Additionally, the rates of perinatal mortality among women with either T1D or T2D are three to four times higher than among the general obstetric population.^4^ Maternal complications include hypertension, haemolysis, pre-eclampsia, elevated liver enzymes, Caesarean section and hypoglycaemia.^5^ A growing body of evidence suggests that greater time in range (TIR), denoting the percentage of time spent in the target blood glucose range of 63–140 mg/dL in pregnancy, is associated with a decreased risk of adverse neonatal outcomes.^6^

Insulin requirements during pregnancy change drastically, with dramatic variations even within a single day, due to shifts in insulin sensitivity.^7^ This necessitates careful monitoring and glycaemic management, especially given that diabetic ketoacidosis in pregnancy is associated with high rates of foetal loss.^8^

Although advanced diabetes technologies, such as automated insulin delivery (AID) systems or hybrid closed-l oop systems, do not have the US Food and Drug Administration (FDA) indications for use in pregnancy, efforts to research AID use in pregnancy are expanding, as continuous glucose monitors (CGMs) alone have shown to be beneficial but insufficient in maintaining optimal glucose control.^9^ The CamAPS FX system, manufactured by CamDiab Ltd based in Cambridge, UK, is the only AID system approved in the UK, EU and USA for use during pregnancy.^10^ The National Institute for Health and Care Excellence has recommended AID for all people with T1D with an HbA1c >8%, including pregnant women with diabetes.^11^ The approval and recommendations are based on a series of trials showing that day-and-night closed-l oop insulin delivery in pregnant women with T1D led to significantly less hypoglycaemia than sensor-augmented pump (SAP) insulin delivery.^12–14^ Moreover, overnight closed-l oop insulin delivery resulted in significantly greater TIR compared with SAP.^13–15^ SAP includes an insulin pump with the use of a CGM, but insulin delivery is not automatically adjusted based on sensor glucose values. Notably, the Automated Insulin Delivery Amongst Pregnant Women With Type 1 Diabetes (AiDAPT; ClinicalTrials.gov Identifier: NCT04938557) trial in pregnant women with T1D, randomized to use the CamAPS FX system or a standard insulin pen or pump with a CGM, showed that participants using the CamAPS FX system spent an additional 2.5 h/day in range.^16^ The use of the CamAPS FX system during pregnancy also improved maternal and neonatal outcomes. Positive maternal outcomes included reduced maternal weight gain and reduced cases of hypertensive disorders, including worsening hypertension, new-onset hypertension and pre-eclampsia. On the neonatal side, there was a significant decrease in gestational age at delivery, along with a smaller percentage of children considered large or extremely large for gestational age. Of note, this system is able to set a glucose target as low as 80 mg/dL, while FDA-approved systems can only be set to a target as low as 100 mg/dL, and many systems have higher targets than that.^16^

Most recently, the use of Medtronic’s (Minneapolis, Minnesota) MiniMed 780G system was shown in the Closed-l oop Insulin Delivery in Pregnant Women With Type 1 Diabetes (CRISTAL; C linicalTrials. gov Identifier: NCT04520971) trial (n=89), presented at the 17th International Conference on Advanced Technologies and Treatments for Diabetes and published in *The Lancet Diabetes & Endocrinology*, to improve overnight TIR while reducing hypoglycaemia in pregnant women with T1D.^17,18^ The trial, however, failed to meet the primary endpoint of overall TIR, which might reflect the cohort’s low baseline HbA1c of 6.5%, as well as the inability to lower the system’s glucose target to under 100 mg/dL. The trial did show a statistically significant improvement in overnight TIR. Due to the algorithm’s meal-assisted technology and ability to subtract insulin even with more intensive carbohydrate ratios, users were recommended to enter additional carbohydrates beyond what they were eating. In findings consistent with the AiDAPT trial, fewer individuals on AID experienced excessive gestational weight gain compared with those on standard care. A smaller observational study evaluating MiniMed 780G in 13 pregnant women with T1D showed a significant increase in TIR and a decrease in HbA1c throughout pregnancy; however, there were high rates of maternal and neonatal complications, with five women having pre-eclampsia and nine women undergoing a Caesarean section.^19^

Prior to the approval of MiniMed 780G and the CRISTAL trial, several case reports demonstrated the successful use of Medtronic’s MiniMed 670G in pregnancy.^20–22^ In one pregnant woman with T1D using MiniMed 670G, Guzmán Gómez et al. found improved glycaemic control, fewer severe hypoglycaemia and hyperglycaemia events and no neonatal or obstetric complications.^22^ Similarly, in another case, after the initiation of MiniMed 670G following the detection of an unplanned pregnancy in a woman with T1D and with a baseline HbA1c level of 7.1%, TIR increased by 4.5%, HbA1c level remained at about 6.5% throughout the pregnancy and no safety events were reported.^20^

Limitations for using AID in pregnancy centre on the stricter glycaemic targets in pregnancy, as current FDA-approved algorithms do not target these glucose levels.^23^ The American Diabetes Association (ADA) defines standard targets in pregnancy as fasting plasma glucose <95 mg/dL and either 1 h postprandial glucose <140 mg/dL or 2 h postprandial glucose <120 mg/dL.^24^ It also recommends an HbA1c target in pregnancy of <6% if it can be achieved without significant hypoglycaemia.

Expert guidance, published last year, offers a framework for off-label use of AID during pregnancy.^25^ The guidance provides several recommendations, including using the lowest target glucose available for the system in use, correcting for hypoglycaemia often but carefully to avoid overcorrection and thus rebound hyperglycaemia, administering extra insulin boluses when needed and adjusting carbohydrate-to-insulin ratios regularly throughout the pregnancy to meet changing insulin needs. Ultimately, while the guidance offers possible pregnancy adjustments for commercially available AID systems, it emphasizes that there is no ‘one-size-fits-all’ approach and calls for individualized medicine, involving flexibility and frequent reassessment.

Accordingly, small studies have investigated pregnancy-specific AID algorithms. In a pilot study, Ozaslan et al. evaluated a zone model predictive control (MPC) algorithm in 11 pregnant women with T1D and showed improved TIR with no serious adverse events.^26^ In another study of a zone MPC algorithm customized for pregnancy in 10 women, Levy et al. showed improved TIR, along with decreases in time above range (TAR) and time below range (TBR). Nine participants achieved >70% TIR.^27^

To better characterize the use of AID in pregnancy, we present a retrospective case review of six pregnant women with T1D who used an AID system off-l abel during pregnancy. This includes two Insulet Omnipod 5 users, two Tandem t:slim X2 Control-I Q users and two Do-I t-Yourself (DIY) or open-source loop users. *Table 1* describes the key characteristics of each system. We describe glycaemic outcomes and insulin requirements throughout the pregnancies.

Omnipod 5, manufactured by Insulet, which is based in Acton, Massachusetts, is an AID system consisting of a wearable, tubeless insulin pump (the Pod), a Dexcom G6 CGM and the Omnipod 5 mobile application available on select smartphones or via a company-provided controller.^28^ Omnipod 5 was first granted FDA approval in January 2022 for people with T1D.^29^ The Pod’s built-i n SmartAdjust technology uses CGM values to increase, decrease or pause insulin delivery every 5 min based on customizable glucose target values (10 mg/dL intervals between 110 and 150 mg/dL). In Automated (Auto) Mode, basal insulin delivery is adjusted based on the current CGM value and its predicted value in 60 min. For exercise or other factors that can predispose to hypoglycaemia, Omnipod 5 has an activity feature, which sets a temporary target glucose to 150 mg/dL, and insulin delivery is less aggressive. This can be set for up to 24 h. Off-l abel use in pregnancy has not been previously reported in the literature.

Tandem Diabetes Care’s (San Diego, California) t:slim X2 Control-I Q hybrid closed loop uses Dexcom G6, Dexcom G7 or Libre 2+ and offers automatic correction boluses up to one every hour and smartphone bolus control.^30^ Control-I Q is the only commercially available AID system with a basal insulin adjustment algorithm that is based on programmed individual basal rates, correction factors and total daily insulin dose, allowing for more customization.^30^ Control-I Q received FDA clearance as class II iController in December 2019 and was also approved as the first ‘interoperable automated glycaemic controller’ (iController), meaning that the algorithm is a lower-risk class II device that can be used with other diabetes devices.^31^ Control-I Q officially launched in January 2020. The Tandem Mobi, which was approved by the FDA in 2023, is compatible with the Dexcom G6 and uses the Control-I Q algorithm. A recent case study of four early adopters of Control-I Q in pregnancy found that the AID system was safely used during pregnancy, birth and postpartum, with participants reporting reduced diabetes management burden and improved sleep.^32^ Participants with low TIR during pregnancy increased their TIR with Control-I Q, and participants with high TIR during pregnancy maintained and increased their TIR. The ongoing Closed-l oop Insulin Delivery In Type 1 Diabetes Pregnancies (CIRCUIT; ClinicalTrials.gov Identifier: NCT04902378) trial is evaluating the Tandem t:slim X2 insulin pump with Control-I Q technology compared with standard insulin delivery plus CGM (n=66).^33^

**Table 1:** An overview of automated insulin delivery systems available in the USA

AID system	Omnipod 5	Control-IQ	DIY Open-Source Loop	MiniMed 670G	MiniMed 770G	MiniMed 780G	iLet
**Company**	Insulet	Tandem	N/A	Medtronic	Medtronic	Medtronic	Beta Bionics
**CGM**	Dexcom G6	Dexcom G6, Dexcom G7 or FreeStyle Libre 2 Plus	Dexcom G6, Dexcom G7, Dexcom ONE, Libre sensors in dev branch only and Medtronic sensors connected to a loop-compatible Medtronic pump	Guardian 3	Guardian 3	Guardian 3 and Guardian 4	Dexcom G6
**Pump**	The Pod	t:slim X2 or Mobi (only compatible with Dexcom G6)	Medtronic 515 or 715 (any firmware), Medtronic 522 or 722 (any firmware), Medtronic 523 or 723 (firmware 2.4 or lower), Medtronic Worldwide Veo 554 or 754 (firmware 2.6A or lower), Medtronic Canadian/Australian Veo 554 or 754 (firmware 2.7A or lower), Omnipod Eros (no longer available in the USA) and Omnipod DASH	MiniMed 670G	MiniMed 770G	MiniMed 780G insulin pump and Medtronic Extended infusion set	iLet ACE Pump
**Algorithm targets**	110, 120, 130, 140 and 150 mg/dL	112.5–160 mg/dL	Referred to as correction range; range of 87–180 mg/dL in master branch; dev branch allows for lower and higher ranges	120 mg/dL	120 mg/dL	100, 110 and 120 mg/dL	110, 120 and 130 mg/dL
**Temporary targets**	Activity: 150 mg/dL	Exercise: 140–160 mg/dL Sleep: 112.5–120 mg/dL	Override: allows temporary target glucose changes as well as %change applied to basal/bolus doses for a defined period (if room allows)	150 mg/dL	150 mg/dL	150 mg/dL	N/A
**Minimum daily insulin**	5 units	10 units	No standard recommended minimum	8 units	8 units	8 units	8 units
**Maximum fill**	200 units	300 units (t:slim X2) or 200 units (Mobi)	Based on the pump in use	300 units	300 units	300 units	180 units
**Basal increment**	0.05 units	0.001 units	Based on the pump in use	0.1, 0.05 or 0.025 units	0.1, 0.05 or 0.025 units	0.1, 0.05 or 0.025 units	N/A
**Bolus increment**	0.05 units	0.01 units	Based on the pump in use	0.1, 0.05 or 0.025 units	0.1, 0.05 or 0.025 units	0.1, 0.05 or 0.025 units	N/A
**Ability to override bolus**	Yes	Yes	Yes	No	No	No	No
**Extended bolus**	No, manual mode only	Yes	No, but it can emulate an extended bolus based on carb absorption	No, manual mode only	No, manual mode only	No, manual mode only	No

*AID = automated insulin delivery; carb = carbohydrate; CGM = continuous glucose monitor; DIY = Do-It-Yourself; N/A = not applicable.*

In addition to these systems, DIY open-source closed-l oop systems have been gaining popularity.^34^ Open-source looping, where target glucose ranges and insulin needs are customizable, is an alternative AID system that involves hacking existing CGMs and pumps to communicate with each other and to connect to a smartphone via a bridging device. OpenAPS and Loop are the two DIY looping open-source options.^35^ They are compatible with a limited number of older pumps. Loop 3, released in 2023, is compatible with Omnipod DASH Pods, while previous versions were only compatible with the Omnipod Eros and older Medtronic pumps.^36^ Loop is developed for iPhone devices, while OpenAPS can run on iPhone and Android devices. Each one takes a different algorithmic approach: Loop uses an MPC algorithm, which predicts future blood glucose levels based on current CGM data and recent insulin activity and adjusts insulin delivery accordingly. OpenAPS uses a proportional-i ntegral-derivative control algorithm, which regulates insulin delivery based on the disparity between current blood glucose levels from a CGM and target ranges.^37^ In January 2023, Tidepool’s Loop (Palo Alto, California) became the first FDA-cleared open-source loop AID algorithm.^38^ Previous case reports of open-source looping in pregnant women have shown reduced diabetes management burden, increased confidence in mitigating hypoglycaemia and achievement of recommended TIR targets.^39^ However, patients have also reported challenges with technical glitches and connectivity issues. Additionally, there is no customer service support line, and there are limited resources for healthcare professionals to use to support their patients. There is a robust online community, with peer support and numerous resources.^40^ A recent case series on four pregnant women with T1D using open-source looping showed that with the exception of the first trimester in the third case, TIR was above 70% throughout the four pregnancies.^41^ There were also no severe hypoglycaemia episodes, although TBR was high in the fourth case.

Omnipod 5, Control-I Q and DIY Open-Source Loop were included in this case review because they are available for use in the USA and are widely used among people with T1D. Due to the timing of FDA approvals and lack of full pregnancy data at this time, the AID systems Beta Bionics’ (Irvine, California) iLet and Medtronic’s 780G are not included in this case series. Tidepool Loop was also excluded because, although it is approved by the FDA, it only recently announced an FDA-approved partner, Sequel MedTech’s (Manchester, New Hampshire) Twiist, which is not yet commercially available. Of the available CGM systems, only Dexcom’s G7 and Abbott’s Libre 2, Libre 2+ and Libre 3 are approved by the FDA for use in pregnancy.^42,43^

## Methods

This retrospective case series included six pregnant women with T1D using three AID systems (*Table 2*): Omnipod 5, Tandem Control-I Q and DIY Loop 3. Electronic medical record data, provider notes and connected data reports were used. Gestational age, system use, average insulin use, TIR, TAR and TBR were collected for each patient at four specified milestones: pre-pregnancy, first trimester, second trimester and third trimester (at the appointment closest to birth). The patient data are from R&B Medical, Tandem t:connect, Glooko (headquartered in Palo Alto, California) and Tidepool (headquartered in Palo Alto, California). We had permission from all respective sites (Cleveland Clinic, University Hospital, Integrated Diabetes Services) to use these databases. The primary outcome was time spent in the target range (60–140 mg/dL), which was adjusted from 63 mg/dL due to limitations of Dexcom Clarity reports. Safety outcomes included incidence of level 1 and level 2 hypoglycaemia, defined as time <63 mg/dL or <54 mg/dL, respectively. Literature references in this article were sourced from publicly available databases, and data on participants in this case series are anonymized and are therefore permitted to be shown. The datasets generated and analysed during the current study are available from authors on reasonable request.

## Results

Across trimesters, the two Omnipod 5 patients had an average TIR of 68 and 82%. Control-I Q patients had an average TIR of 77 and 69%. Both DIY Loop patients had an average TIR of 85% (*Table 3*). Hypoglycaemia occurrence was minimal (*Table 4*). There were no occurrences of severe hypoglycaemia or diabetic ketoacidosis. Additionally, four of the six patients had uncomplicated vaginal deliveries in their third trimester. Birth complications for the other two patients were resolved shortly after birth (*Table 5*).

**Table 2:** Pre-pregnancy baseline characteristics

AID system and patient	Comorbid conditions	Age at diagnosis (years)	Age at delivery (years)	Height (inches)	Body weight (kg)	Average glucose (mg/dL), SD	GMI (%)	TDD insulin (units)	Basal insulin (units)	Bolus insulin (units)	Carbs entered/day (g)
Omnipod 5 patient 1	Obesity and hypertension	6	39	62.5	100	179, 62	8.3	46.8	28.6	18.2	223
Omnipod 5 patient 2	None	10	34	64	62	123, 46	6.3	32.5	16.1	16.4	142
Control-I Q patient 1	Hypothyroidism and retinopathy	11	29	59	49	162, 33	7.2	25.5	16.4	4.32 0.75 correction bolus 3.98 autobolus	104
Control-I Q patient 2	None	9	28	68.5	80	155, 65	7	37.6	17.6	14.4 3.2 correction bolus 1.8 autobolus	170
DIY Loop patient 1	None	12	35	64	54	109, 32	5.9	43.1	Loop delivery: 12.6 Base basal: 15.5	30.5	220
DIY Loop patient 2	Hypothyroidism	4	28	62	52	107, 33	5.9	31.2	Loop delivery: 7.8 Base basal: 14.85	23.4 (includes autobolus)	100

*AID = automated insulin delivery; carbs = carbohydrates; DIY = Do-It-Yourself; GMI = glucose management indicator; SD = standard deviation; TDD = total daily dose.*

**Table 3:** Time in range data 60–140 mg/dL

AID system and patient	First trimester (%)	Second trimester (%)	Third trimester (%)	Average (%)
Omnipod 5 patient 1	64	72	68	68
Omnipod 5 patient 2	74	88	84	82
Control-I Q patient 1	77	74	81	77
Control-I Q patient 2	64	61	81	69
DIY Loop patient 1	87	80	89	85
DIY Loop patient 2	82	84	89	85

*AID = automated insulin delivery; DIY = Do-It-Yourself.*

### Omnipod 5

#### Omnipod 5 patient 1

Patient 1 using Omnipod 5 was aged 39 years at conception, with a 33-year history of T1D. Comorbid conditions included hypertension, treated with labetalol and obesity. Her pre-pregnancy body weight was 100 kg, and she gained 17 kg over the pregnancy. She also developed pre-eclampsia. She delivered via Caesarean section at 36 1/7 weeks with a 48 h neonatal intensive care unit (NICU) admission for hypoglycaemia (42 mg/dL) at 6 h after delivery. Her previous pregnancy without AID resulted in delivery at 31 weeks, hypoglycaemia and a 4-week NICU admission.

#### Glycaemic outcomes

The patient was using Omnipod 5 pre-pregnancy with a glucose management indicator (GMI) of 8.3%, average glucose of 179 mg/dL and TIR of 54%. Her TIR improved throughout the pregnancy and was highest (72%) at the end of the second trimester. At delivery, her TIR was 68%, and she had an average glucose of 119 mg/dL. Her GMI also improved throughout the pregnancy, decreasing each trimester to 6.6% at delivery (*Table 6*). Notably, her fasting glucose ranged from 110 to 115 mg/dL while using Omnipod 5 in Auto Mode overnight. While this did not meet the ADA’s fasting plasma glucose target of <95 mg/dL, overall TIR still improved, and she did not have the burden of having to use manual mode overnight.

#### Insulin requirements

Her total daily dose (TDD) insulin needs increased from 46.8 to 72.2 units/day by the end of the first trimester. By the end of the second trimester, she needed 94.7 units/day. By the time of her delivery, she needed 122.2 units/day, which is an overall increase of 161%. The Omnipod 5 pump can hold up to 200 units, so she needed more frequent pump changes as the pregnancy progressed.

#### Omnipod 5 patient 2

Patient 2 using Omnipod 5 was aged 34 years at conception with a 24-year history of T1D and no comorbid conditions. Her pre-pregnancy body weight was 62 kg, and she gained 9 kg over the pregnancy. She delivered at 39 weeks via a spontaneous vaginal delivery, and she did not experience complications.

#### Glycaemic outcomes

The patient had a pre-pregnancy GMI of 6.3%, average glucose of 123 mg/dL and TIR of 74%. Her TIR improved throughout the pregnancy and was the highest (88%) at the end of the second trimester. At delivery, TIR was 84%, and she had an average glucose of 108 mg/dL. Her GMI also improved throughout the pregnancy, decreasing to 6.1% at delivery. Notably, for this patient, Auto Mode was turned off overnight during the pregnancy. To treat hyperglycaemia that occurred when in manual mode, the patient was instructed to bolus for ‘fake carbohydrates’ or carbohydrates she did not consume.

#### Insulin requirements

Insulin needs increased from an initial TDD of 32.5 to 37.6 units/day by the end of the first trimester. By the end of the second trimester, she needed 41.4 units/day. By the time of her delivery, she needed 58.9 units/day, which is an overall increase of 81%. She did limit her carbohydrate consumption to 100 g/day in the second trimester and 142 g/day in the third trimester, and this may explain the reduced expected insulin increase for pregnancy.

#### Delivery

For both patients using the Omnipod 5, the pump was reset due to concerns of significantly decreasing insulin needs at the time of delivery – the Omnipod 5 uses adaptive basal rates based on the previous Pods’ total daily dose, with the last four to five Pods having the greatest effect in the declining total daily insulin-weighted average. Pre-pregnancy settings were entered, and Omnipod 5 was reset, which restarts the algorithm. Neither person developed hypoglycaemia post-delivery. Another option would have been to leave the person in the manual mode with pre-pregnancy dose settings for four to five Pod changes.

**Table 4:** Time below range data <60 and <54 mg/dL

AID system and patient	First trimester (%)	Second trimester (%)	Third trimester (%)	Average (%)
Omnipod 5 patient 1	3, 2	1, 2	3, 2	2, 2
Omnipod 5 patient 2	2, 1	3, 2	2, 1	2, 1
Control-I Q patient 1	<1, <1	0, 0	0, 0	<1, 0
Control-I Q patient 2	2, 0	3, 0	3, 0	3, 0
DIY Loop patient 1	3, 0	3, <1	3, 0	3, 0
DIY Loop patient 2	1, <1	2, <1	<1, <1	1, <1

*AID = automated insulin delivery; DIY = Do-It-Yourself.*

**Table 5:** Pregnancy outcomes

AID system and patient	Baby’s weight (kg)	Delivery method
Omnipod 5 patient 1	3.946	Caesarean section at 36 1/7 weeks due to pre-eclampsia, 48 h NICU admission due to hypoglycaemia (42 mg/dL) at 6 h after delivery
Omnipod 5 patient 2	3.311	Delivery at 39 weeks of gestation Spontaneous vaginal delivery and no complications
Control-I Q patient 1	3.033	Preterm premature rupture of membranes led to early delivery at 34 2/7 weeks of gestation, vaginal delivery and no complications
Control-I Q patient 2	3.650	Caesarean section at 37 2/7 weeks due to gestational hypertension requiring parenteral nifedipine and resolved with delivery of placenta without further complications
DIY Loop patient 1	3.345	Delivery at 39 5/7 weeks, vaginal delivery and no complications (baby with tongue tie)
DIY Loop patient 2	3.963	Delivery at 37 6/7 weeks, vaginal delivery and no complications

*AID = automated insulin delivery; DIY = Do-It-Yourself; h = hours; NICU = neonatal intensive care unit.*

### Control-IQ

#### Control-IQ patient 1

Patient 1 using Control-I Q was aged 29 years at conception, with an 18-year history of T1D. Comorbid conditions included hyperthyroidism and retinopathy. Her pre-pregnancy body weight was 49 kg, and she gained 10 kg over the pregnancy. The baby was delivered early without complications via vaginal birth at 34 2/7 weeks due to premature rupture of membranes. The baby required a 2-week NICU stay for further lung development.

#### Glycaemic outcomes

The patient was using Control-I Q pre-pregnancy, with a GMI of 7.2%, average glucose of 162 mg/dL and TIR of 60%. She used the sleep mode throughout the entire pregnancy, which has a tighter target of 112.5–120 mg/dL. Her TIR was consistently over 70% throughout the pregnancy, with limited hypoglycaemia. TIR was highest in the third trimester (81%). Her GMI also improved throughout her pregnancy to 6.2% in the third trimester.

#### Insulin requirements

The patient’s TDD decreased from 25.5 units/day pre-pregnancy to 21.7 units/day in the first trimester. In the second trimester, her insulin needs increased to 33.5 units/day, and by the third trimester, her insulin needs increased slightly further to 34.5 units/day for an overall increase of 35%, which is below the two-to threefold increase usually observed in pregnancy.

#### Control-IQ patient 2

Patient 2 using Control-I Q was aged 28 years at conception, with a 19-year history of T1D and no comorbid conditions. Her pre-pregnancy body weight was 80 kg, and she gained 11 kg over the pregnancy. The baby was delivered early without complications via Caesarean section at 37 2/7 weeks due to gestational hypertension requiring parenteral nifedipine. Control-I Q was completely stopped in the third trimester and lower glucose levels were achieved with a corresponding higher TIR via manual settings.

#### Glycaemic outcomes

The patient had been on an insulin pump since 2005 and on Tandem Control-I Q since 2020. She had a pre-pregnancy GMI of 7%, average glucose of 155 mg/dL and TIR of 71%. TIR decreased to 64% in the first trimester and 61% in the second trimester, but it increased to 81% in the third trimester. Her GMI decreased throughout the pregnancy and reached 6.0% in the third trimester.

#### Insulin requirements

Carbohydrate ratios were intensified. Because the active insulin time is set at 5 h in an automated mode, the correction factor was intensified to be more aggressive, but approximately 10% override doses or fake carbohydrates were still often needed to correct high glucose. The patient’s TDD increased from 37.6 units/day pre-pregnancy to 53.5 units/day in the first trimester. In the second trimester, her insulin needs increased to 60.35 units/day, and by the third trimester her insulin needs increased further to 70.96 units/day, which is an overall increase of 88%.

### Do-It-Yourself Loop 3

#### Do-It-Yourself Loop 3 patient 1

Patient 1 using DIY Loop 3 was aged 35 years at conception, with a 23-year history of T1D and no comorbid conditions. Her pre-pregnancy body weight was 54 kg and she gained 8 kg over the pregnancy. She delivered via vaginal birth at 39 5/7 weeks. The baby had no episodes of hypoglycaemia post-delivery and was treated after discharge for tongue tie.

**Table 6:** Average glucose and glucose management indicator (GMI) throughout pregnancy

	First trimester	Second trimester	Third trimester
AID system and patient				Average glucose (mg/dL), SD	GMI (%)	Average glucose (mg/dL), SD	GMI (%)	Average glucose (mg/dL), SD	GMI (%)
Omnipod 5 patient 1	138, 46	7.3	112, 39	6.7	119, 36	6.6
Omnipod 5 patient 2	116, 43	6.1	112, 26	6.1	108, 43	6.1
Control-I Q patient 1	117, 26.8	6.1	127, 28	6.4	119, 28	6.2
Control-I Q patient 2	138, 43	6.3	133, 25	6.5	110, 33	6.0
DIY Loop patient 1	88, 25	5.5	112, 32	6.0	96, 24	5.6
DIY Loop patient 2	109, 33	5.9	107, 28	5.9	105, 27	5.9

*AID = automated insulin delivery; DIY = Do-It-Yourself; GMI = glucose management indicator; SD = standard deviation.*

**Table 7:** Time above range data >140 mg/dL

AID system and patient	First trimester (%)	Second trimester (%)	Third trimester (%)	Average (%)
Omnipod 5 patient 1	28	24	26	26
Omnipod 5 patient 2	20	6	13	13
Control-I Q patient 1	20	26	19	22
Control-I Q patient 2	34	36	16	29
DIY Loop patient 1	10	17	4	10
DIY Loop patient 2	15	13	9	12

*AID = automated insulin delivery; DIY = Do-It-Yourself.*

#### Glycaemic outcomes

Just prior to conception, the patient was using Omnipod 5 with a GMI of 5.9%, average glucose of 109 mg/dL and TIR of 84%. During her pregnancy, she was using DIY Loop. She used an automatic bolus strategy throughout the entire pregnancy with the target range of 85–95 mg/dL. Her TIR was consistently over 80% throughout the pregnancy with limited hypoglycaemia. Overall, her TIR and GMI improved throughout the pregnancy. At delivery, her TIR was 87% and her GMI was 5.6%. Notably, her TIR was lowest in the second trimester (80%), and her GMI was also the highest in that trimester (6%). This was primarily due to more TAR (17% >140 mg/dL) (*Table 7*), which decreased to just 4% at delivery, the lowest throughout the pregnancy and pre-pregnancy.

#### Insulin requirements

Her TDD insulin needs decreased from 43.1 units/day pre-pregnancy (on Omnipod 5) to 29.7 units in the first trimester (now on DIY Loop). Her insulin needs increased to 44.5 units/day in the second trimester and to 60.5 units/day by the end of the third trimester, for an overall increase of 40%.

#### Do-It-Yourself Loop 3 patient 2

Patient 2 using DIY Loop 3 was aged 28 years at conception, with a 24-year history of T1D. The patient also had hypothyroidism treated with levothyroxine. Her pre-pregnancy body weight was 52 kg, and she gained 14 kg over the pregnancy. She delivered via vaginal birth at 37 6/7 weeks with no complications.

#### Glycaemic outcomes

The patient had a pre-pregnancy GMI of 5.9%, average glucose of 107 mg/dL and TIR of 81%. She used an automatic bolus strategy throughout the entire pregnancy, with the target range of 80–90 mg/dL from 10 pm to 6 am and 90–100 mg/dL from 6 am to 10 pm. Her TIR improved throughout the pregnancy, with limited hypoglycaemia, and was highest (89%) at the end of the third trimester. Her GMI remained consistent throughout the pregnancy.

#### Insulin requirements

Insulin needs decreased from an initial TDD of 31.2 to 30.6 units/day by the end of the first trimester. By the end of the second trimester, she needed 43.5 units/day. By the time of her delivery, she needed 54.4 units/day, which is an overall increase of 74%.

## Discussion

These six case studies demonstrate the successful use of three different AID systems during pregnancy. In all cases, insulin requirements (*Table 8*) and weight increased throughout pregnancy as expected. Carbohydrate ratios and correction factors were intensified, and basal rates were increased with all systems, although there were some important differences in the management with the different respective systems. See *Table 9* for general considerations when using AID systems in pregnancy. While CGM use provided granular information about patients’ glycaemic management throughout pregnancy, these studies are limited by the absence of finger stick data that could have assessed the accuracy of CGM values.

**Table 8:** Insulin use throughout pregnancy

	First trimester	Second trimester	Third trimester
AID system and patient				TDD insulin Basal insulin (units)	Bolus insulin (units)	TDD insulin Basal insulin (units)	Bolus insulin (units)	TDD insulin Basal insulin (units)	Bolus insulin (units)
Omnipod 5 patient 1	72.2	39.9	32.3	94.7	49.2	45.5	122.2	55.8	66.4
Omnipod 5 patient 2	37.6	18.6	19.0	41.4	17.4	24.0	58.9	26.5	33.3
Control-I Q patient 1	21.7	12.2	7.7 1.83 correction bolus	33.5	16.06	16.45 0.98 correction bolus	34.5	14.0	20.05 0.48 correction bolus
Control-I Q patient 2	53.5	20.08	30.98 1.33 correction bolus	60.35	23.9	36.05 0.4 correction bolus	71.0	25.2	42.2 3.56 correction bolus
DIY Loop patient 1	29.7	Loop delivery: 12.3 Base basal: 17.05	17.4	44.5	Loop delivery: 12.1 Base basal 17.1	32.4 (includes autobolus)	60.5	Loop delivery: 13.8 Base basal: 24.8	46.7 (includes autobolus)
DIY Loop patient 2	30.6	Loop delivery: 7.9 Base basal: 14.7	22.7 (includes autobolus)	43.5	Loop delivery: 11.4 Base basal: 17.0	32.1 (includes autobolus)	54.4	Loop delivery: 15.2 Base basal: 20.7	39.2 (includes autobolus)

*AID = automated insulin delivery; DIY = Do-It-Yourself; TDD = total daily dose.*

**Table 9:** Pregnancy considerations with automated insulin delivery systems

Component	Consideration
Carbohydrate ratios	Will need to be intensified (lowered) throughout the second and third trimesters
Correction factors	Will need to be intensified (lowered) throughout the second and third trimesters
Basal rates	May not affect the algorithm, however should be increased throughout pregnancy to match automated basal rates in case of limited or defaulting to the manual mode
Insulin action time	If adjustable, shorter times are typically needed (2–3 h)
BG target	Generally set as low as the system will allow
Correction doses	Many additional correction doses are likely to be needed, especially postprandially, in addition to any automated corrections delivered by the system
Glucose ranges	Target range is 63–140 mg/dL; however, some systems will require you to round to 65–140 or 60–140 mg/dL
Delivery	Change settings to pre-pregnancy and may benefit from resetting the pump (Omnipod 5) or spending some time manually before resuming automation if it is an algorithm based on TDI

*BG = blood glucose; TDI = total daily insulin dose.*

To date, there have been extremely few randomized controlled trials for AID use in pregnant women. The Continuous Glucose Monitoring in Women With Type 1 Diabetes in Pregnancy Trial (CONCEPTT; ClinicalTrials.gov Identifier: NCT01788527) trial (n=325) set the stage for the use of CGM in pregnancy by demonstrating that CGM improves maternal glycaemia and gestational health outcomes.^9,44^

In the context of the relative dearth of research on AID use in pregnancy, the findings from this case series point to a promising role for incorporating automated systems into diabetes care for pregnant women. Indeed, five out of six participants in this case series achieved a greater average TIR throughout pregnancy than the 68% average TIR achieved by participants on CGM in the CONCEPTT trial, suggesting the potential of added benefit with AID use. Similarly, the CamAPS FX group in the AiDAPT trial also spent, on average, 68% TIR. The greater TIR achieved by participants in this case series reflects the potential of other AID systems to achieve even further improved outcomes, as well as the importance of investigating AID use in pregnancy in more randomized controlled trials.

### Omnipod 5

Results for the Omnipod users suggest that turning off Auto Mode overnight may be helpful to achieve the ADA fasting glucose target of less than 95 mg/dL.^45^ However, this decision should be made on an individual basis, as the lowest target that can be set by the system is 110 mg/dL. In addition, prior to delivery, the pump was reset to align with pre-pregnancy settings, consistent with the decreasing insulin needs at the time of delivery. With these modifications, neither patient developed hypoglycaemia following delivery. The algorithm of this system uses an adaptive basal rate, which is based on TDD, and has certain restrictions on how high the adaptivity can go. In general, people can achieve lower mean glucose and higher TIR with more frequent boluses for correction and a higher bolus-to-basal ratio.^46^ Although basal rates do not affect the automatic basal delivery, if sensor glucose is lost for more than 20 min, the system can go into a limited mode where it will default to the adaptive basal rate or the programmed basal rate, whichever is lower. In the manual mode, the system would work off the programmed basal rates. Therefore, basal rates are still adjusted throughout pregnancy to represent the person’s current needs. Of note, this pump only holds 200 units, requiring more frequent Pod changes as pregnancy progresses. Some people use U200 insulin, which is twice as concentrated, and allows the pump to hold 400 units/day. This, however, requires manual calculations of carbohydrate ratios and correction factors.

### Control-IQ

Meanwhile, for both patients using Tandem Control-I Q, carbohydrate ratios were intensified to achieve better glycaemic control. Specifically, the correction factor was intensified to be more aggressive, and override doses or fake carbohydrates were still needed to correct high glucose, as even in sleep mode, the target is set for 112.5–120 mg/dL. One factor that helped to optimize the system was setting higher basal rates, which are factored into the basal delivery and often allowed for lower mean glucose to be achieved overnight. Of note, the insulin action time cannot be modified with this system. However, if basal rates are set higher, and the pump ends up delivering less insulin than the programmed basal rate, this will allow for less insulin on board and can lead to the ability to give more aggressive correction doses. This pump holds 300 units of insulin, making it more likely to last the full 2–3 days of recommended wear time.

### Do-It-Yourself Loop 3

With Loop, the adjustable features of the algorithm allowed both patients to achieve over 80% TIR and minimal hypoglycaemia during each trimester. Changes to insulin doses in basal rates, carbohydrate rates and correction factors enabled the algorithm to respond to changes in glucose over the course of pregnancy. Loop accommodates meals by following the absorption time entered with the carbohydrate grams.^47^ This allows Loop to follow the effect of this meal and evaluate the change in glucose relative to carbohydrates on board and insulin on board. As digestion, insulin needs and tolerance to carbohydrate load shift during pregnancy, Loop’s functionality is beneficial in maintaining postprandial glycaemic goals. Additional features such as ‘override’ allow for situational customization around activity, stress and illness. Overrides were used in both pregnancies as insulin needs to be shifted up to adjust basal and bolus doses. Loop also allows tracking of non-pumped insulin, which ensures that injected insulin is factored into the insulin on board when the system makes algorithmic adjustments. As insulin sites get heavily saturated due to the increase in total dose, heavy boluses may be challenging. During the third trimester, both patients needed to use injections for doses that were larger than 8 units at one time. Loop can account for the injected dose, along with the pumped insulin.^48^

## Conclusion

In this case series, women with T1D successfully used three different AID systems during pregnancy. Although these AID systems are not approved by the FDA for pregnancy and two out of three lack pregnancy-specific glucose targets, patients were able to achieve near-desired targets in pregnancy with minimal hypoglycaemia. They also had similar or higher average TIRs compared with landmark pregnancy trials with diabetes technology, such as CONCEPTT and AiDAPT. Four out of six patients met the guideline-recommended goals for TIR. On average, insulin requirements doubled throughout the pregnancies. Of note, heavy boluses may be challenging with DIY Loop, and patients may need additional injections for doses. Randomized controlled trials with these systems are needed to gain FDA approval for use during pregnancy. In the meantime, real-world data and experience are showing that these systems can be successfully used in pregnancy and may even be superior to multiple daily injections or pump use without automation.
